# Diagnosing Kaposi’s Sarcoma (KS) in East Africa: how accurate are clinicians and pathologists?

**DOI:** 10.1186/1750-9378-7-S1-P6

**Published:** 2012-04-19

**Authors:** Erin Amerson, Nathan Buziba, Henry Wabinga, Megan Wenger, Mwebesa Bwana, Winnie Muyindike, Catherine Kyakwera, Miriam Laker, Edward Mbidde, Constantin Yiannoutsos, Kara Wools-Kaloustian, Beverly Musick, Philip LeBoit, Tim McCalmont, Beth Ruben, Paul Volberding, Toby Maurer, Jeffrey Martin

**Affiliations:** 1 University of California, San Francisco, USA; 2 Moi University, Eldoret, Kenya; 3 Makerere University, Kampala, Uganda; 4 Mbarara University of Science and Technology, Mbarara, Uganda; 5 Infectious Diseases Institute, Kampala, Uganda; 6 Indiana University, Indianapolis, IN, USA

## Background

HIV-associated KS is the most common reported malignancy in sub-Saharan Africa, and appropriate therapy of KS requires accurate diagnosis. In much of the region, however, KS diagnosis is limited to clinical suspicion without pathologic confirmation. Where pathology is available, specific anti-KSHV stains are rarely available and overall pathologic accuracy for KS has not been evaluated.

## Methods

We introduced skin punch biopsy for KS at HIV/AIDS care clinics in Uganda and Kenya within the East Africa IeDEA Consortium. Clinicians suspecting KS could obtain a biopsy same day without charge. After interpretation by local African pathologists who only had access to routine H&E staining, biopsies were read by dermatopathologists at UCSF who could, at their discretion, recut and restain specimens or stain against latency-associated nuclear antigen (LANA) of KSHV. The interpretation by the U.S. dermatopathologists, who serve a large base of HIV-infected patients, was considered the gold standard.

## Results

Clinicians at 26 HIV/AIDS clinics in Uganda and Kenya referred 739 patients with clinically suspected KS for skin biopsy. Overall, 77% (95% CI: 74%--80%) of these clinically suspected cases were determined pathologically to be KS after U.S. review; 19% had another diagnosis and 4% were indeterminate. There was no significant difference in the percentage found to be KS between countries (p=0.20) or over time (p=0.11). When KS was not found, a wide variety of other diagnoses, both clinically significant and insignificant, were made by the U.S. dermatopathologists (Table 1). Two different pathology services, one in Uganda one in Kenya, submitted biopsies for review by U.S. dermatopathologists. Overall concordance between African and U.S. interpretations was 71% (95% CI: 68%--74%). When the U.S. interpretation was considered gold standard, sensitivity of the African pathologic interpretation for KS was 72% and specificity 84%. Over time, sensitivity increased at one African center (p=0.04) but decreased in another (p<0.001); specificity increased at one center (p=0.001) and was unchanged in another (p=0.68).

**Table 1:** 

Sample of pathologic diagnoses made by U.S. dermatopathologists when KS was not present			
Scar (n=9)	Post-inflammatory pigmentation (9)	Psoriasis (8)	Lymphoma (5)

Wart (5)	Bacillary angiomatosis (4)	Morphea (4)	Sarcoidosis (2)

Polyarteritis nodosa (2)	Pyogenic granuloma (2)	Mycobacterial dermatitis (2)	Lichen planus (2)

Dermatofibroma (2)	Castleman’s Disease (1)	Squamous cell carcinoma (1)	Deep fungal infection (1)

Secondary syphilis (1)	Erythema multiforme (1)	Eccrine poroma (1)	Xanthoma (1)

## Conclusions

Amongst clinicians at HIV/AIDS clinics in East Africa, clinical suspicion of KS alone is not optimally specific for KS diagnosis. Clinical suspicion alone often either misdiagnoses conditions which are less concerning than KS or misses other serious conditions that require different therapy than KS. Assuming the U.S. interpretation is the gold standard, pathologic determination of KS in East Africa is specific but not optimally sensitive. The findings urge for increased availability of skin punch biopsies for KS diagnosis in Africa and augmentation of pathology services.

